# Supplementary material for: Viroinformatics-Based Analysis of SARS-CoV-2 Core Proteins for Potential Therapeutic Targets
Source: Antibodies (Basel). 2021 Jan 11;10(1):3. doi: 10.3390/antib10010003 (PMC7839017; doi:10.3390/antib10010003)
Supplement: Supplementary file 1 [file antibodies-10-00003-s001.pdf]

# Supplementary Tables

**Table S1:** List of the ten top hits of antiviral drugs against SARS-CoV-2 Spike protein by order of lowest to highest binding energy value. Indinavir seems to exhibit the lowest binding energy (-9.8 kcal/Mol) when docked with Spike protein, whereas Cobicistat exhibits the highest binding energy (-6.37 kcal/Mol).

| | **Antivirals** | **Binding energy (kcal/Mol)** | **2D interaction** | **Interface amino acids** | **Type of interactions** |
|---|---|---|---|---|---|
| 1 | Indinavir (C36H47N5O4), 5362440 | -9.8 | 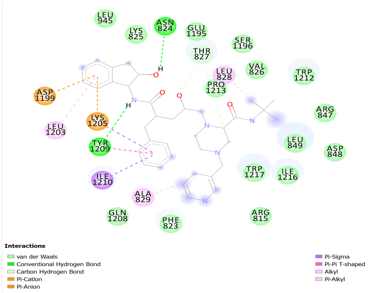 | Ala, 2Arg, Asn, 2Asp, Gln, Glu, 2Ile, 4Leu, 2Lys, Phe, Pro, Ser, Thr, 2Trp, Tyr | 1Alk, 1CHB, 2HB, 1Pi-An, 3Pi-Alk, 1Pi-Cat, 2Pi-Sig,1Pi-Pi-T, 14VW |
| 2 | Nelfinavir (C32H45N3O4S), 64143 | -9 | 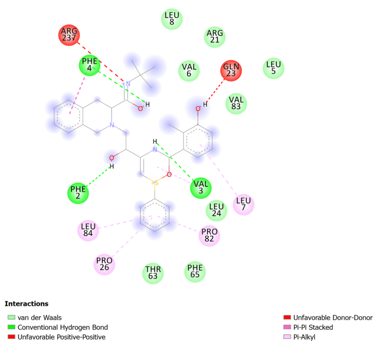 | 2Arg, Gln, 5Leu, 3Phe, 2Pro, Thr, 3Val | 3HB, 5Pi-Alk, Pi-Pi-Stk, 7VW, 1UDD, 1UPP |
| 3 | Fosamprenavir (C25H36N3O9PS), 131536 | -8.2 | 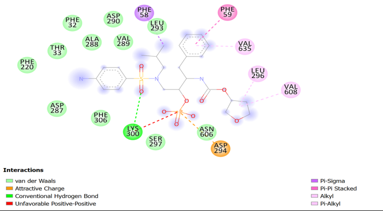 | Ala, Asn, 3Asp, 5Phe, 2Leu, Lys, Ser, 3Val | 1AC, 2Alk, 1HB, 1Pi-Alk, 1Pi-Pi-Stk, 1Pi-Sig, 1UPP, 11VW |
| 4 | Rintatolimod (C28H40N9O25P3), 135537060 | -7.6 | | Arg, 3Asn, 2Asp, 2Gln, 2Glu, Gly, 2Ile, 2Lys, Pro, 2Ser, 1Val | 1AC, 1CHB, 5HB, 14VW |

| | | | | | |
|---|---|---|---|---|---|
| | | | 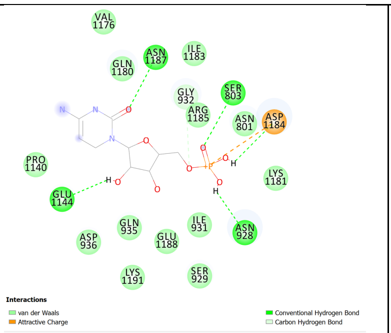 | | |
| 5 | Loviride ($C_{17}H_{16}Cl_2N_2O_2$), 3963 | -7.3 | 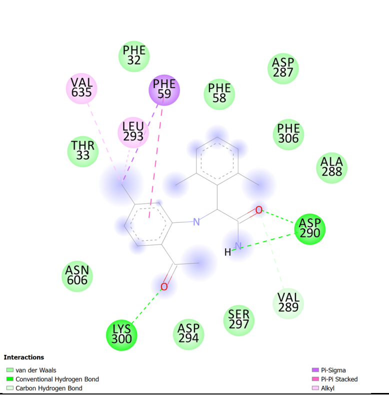 | Ala, Asn, 3Asp, Leu, Lys, 4Phe, Ser, Thr, 2Val | 2Alk, 1CHB, 3HB, 1Pi-Pi-Stk, 1Pi-Sig, 9VW |
| 6 | Nevirapine ($C_{15}H_{14}N_4O$), 4463 | -7.3 | 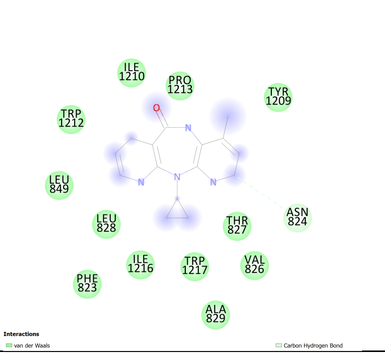 | Ala, Asn, 2Ile, 2Leu, Phe, Pro, Thr, 2Trp, Tyr, Val | 1CHB,12VW |
| 7 | Nitazoxanide ($C_{12}H_9N_3O_5S$), 41684 | -7.1 | 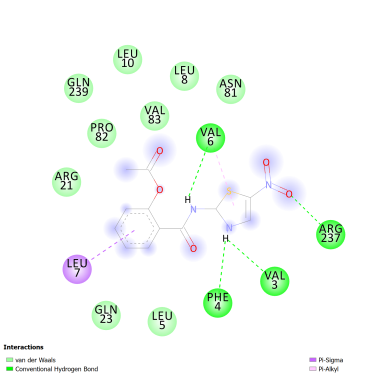 | 2Arg, Asn, 2Gln, Phe, Pro, 4Leu, 3Val | 4HB,12Pi-Alk, 1Pi-Sig, 9VW |
| 8 | Imiquimod ($C_{14}H_{16}N_4$), 57469 | -6.8 | | Asn, Ile, 2Leu, Lys, Phe, Pro, Thr, Trp, Tyr, Val | 2Alk, 2HB, 1Pi-Alk, 5VW |

| | | | | | |
|---|---|---|---|---|---|
| | | | 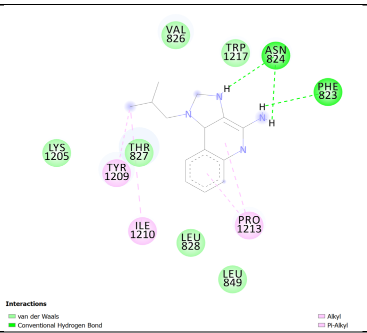 | | |
| 9 | Inosine (C10H12N4O5), 135398641 | -6.5 | 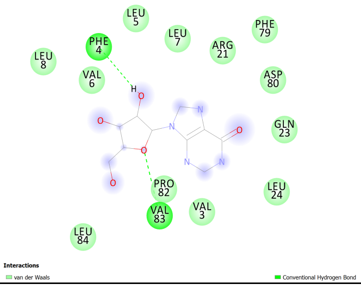 | Arg, Asp, 5Leu, 2Phe, Pro, 3Val | 2HB, 12VW |
| 10 | Cobicistat (C40H53N7O5S2), 25151504 | -6.37 | 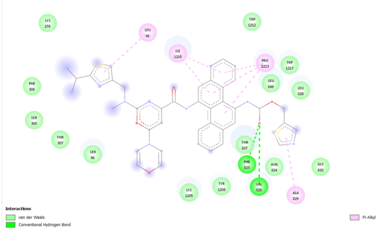 | 1Ala, 1Asn, 1Gly, 1Ile, 3Leu, 2Lys, 2Phe, 1Pro, 2Ser, 2Thr, 2Trp, 1Tyr, 1Val | 2HB, 7Pi-Alk, 14VW |

**Table S2:** List of the ten top hits of antibiotics drugs against SARS-CoV-2 Spike protein by order of lowest to highest binding energy value. Vancomycin seems to exhibit the lowest binding energy (-10.2 kcal/Mol) when docked with Spike protein, whereas Levofloxacin exhibits the highest binding energy (-5.11 kcal/Mol).

| | **Antibiotics** | **Binding energy (kcal/ Mol)** | **2D interaction** | **Interface amino acids** | **Type of interactions** |
|---|---|---|---|---|---|
| 1 | Vancomycin ($C_{66}H_{75}Cl_2N_9O_{24}$), 14969 | -10.2 | 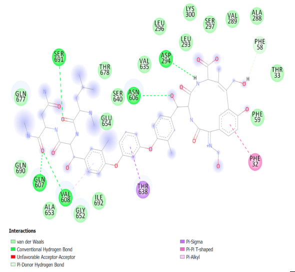 | 2Ala, Asn, Asp, 3Gln, Glu, Gly, Ile, 2Leu, Lys, 3Phe, 3Ser, 3Thr, 2Val | 5HB, 1Pi-Alk, 1Pi-DHB, 1Pi-Pi-T, 1Pi-Sig, 17VW |
| 2 | Gliclazide ($C_{15}H_{21}N_3O_3S$), 3475 | -8.6 | 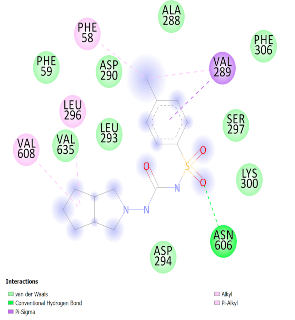 | Ala, Asn, 2Asp, 2Leu, Lys, 3Phe, Ser, 2Val | 3Alk, 1HB, 1Pi-Alk, 1Pi-Sig, 9VW |
| 3 | Azithromycin ($C_{38}H_{72}N_2O_{12}$), 447043 | -7.9 | 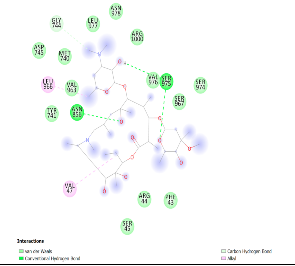 | 2Arg, 2Asn, Asp, Gly, 2Leu, Met, Phe, 4Ser, Tyr, 3Val | 2Alk, 1CHB, 3HB, 13VW |
| 4 | Sulfamethoxazole ($C_{10}H_{11}N_3O_3S$), 5329 | -6.6 | 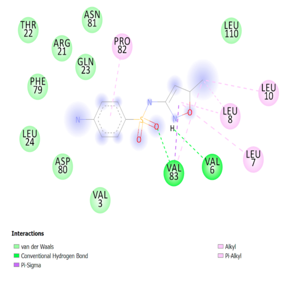 | Arg, Asn, Asp, Gln, 5Leu, Phe, Pro, Thr, 3Val | 3Alk, 2HB, 3Pi-Alk, 1Pi-Sig, 9VW |

| | | | | | |
|---|---|---|---|---|---|
| 5 | Meropenem ($C_{17}H_{25}N_3O_5S$), 441130 | -6.4 | 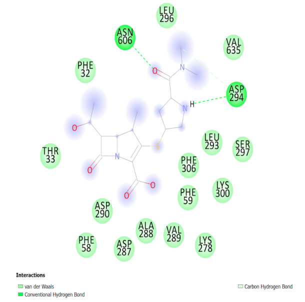 | Ala, Asn, 3Asp, 2Leu, 2Lys, 4Phe, Ser, Thr, 2Val | 1CHB, 2HB, 15VW |
| 6 | Tenofovir Disoproxil ($C_{19}H_{30}N_5O_{10}P$), 5481350 | -6.3 | 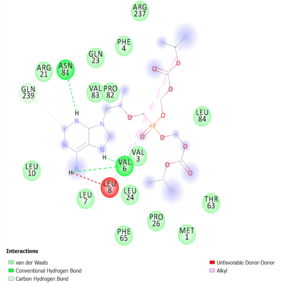 | 2Arg, Asn, 2Gln, 5Leu, Met, 2Phe, 2Pro, Thr, 3Val | 2Alk, 1CHB, 2HB, 1UDD, 16VW |
| 7 | Trimethoprim ($C_{14}H_{18}N_4O_3$), 5578 | -6.1 | 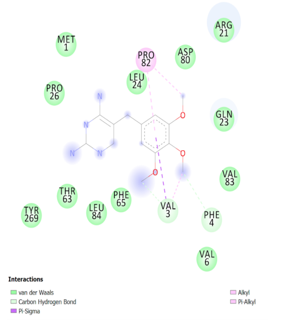 | Arg, Asp, Gln, 2Leu, Met, 2Phe, 2Pro, Thr, Tyr, 3Val | 2Alk, 2CHB, 1Pi-Alk, 1Pi-Sig, 12VW |
| 8 | Ciprofloxacin ($C_{17}H_{18}FN_3O_3$), 2764 | -5.58 | 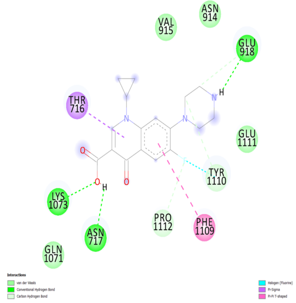 | 2Asn, 1Gln, 2Glu, 1Lys, 1Phe, 1Pro, 1Thr, 1Tyr, 1Val | 3CHB, 1Hal, 3HB, 1Pi-Pi-T, 1Pi-Sig, 4VW |
| 9 | Gentamicin ($C_{21}H_{43}N_5O_7$), 3467 | -5.4 | 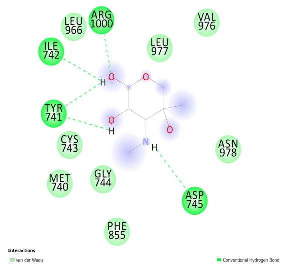 | Arg, Asn, Asp, Cys, Gly, Ile, 2Leu, Met, Phe,Tyr, Val | 5HB, 8VW |
| 10 | Levofloxacin ($C_{18}H_{20}FN_3O_4$), 149096 | -5.11 | 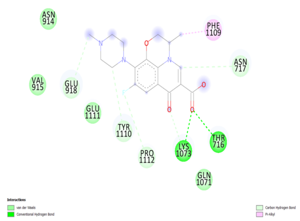 | 2Asn, 1Gn, 2Glu, 1Lys, 1Phe, 1Pro, 1Thr, 1Tyr, 1Val | 5CHB, 2HB, 1Pi-Alk, 4VW |

**Table S3:** List of the top hits antiparasitic drugs, flavonoids, and Vitamins against SARS-CoV-2 Spike protein by order of lowest to highest binding energy value. Ivermectin B1a seems to exhibit the lowest binding energy (-9.16 kcal/Mol) when docked with Spike protein, whereas Hydroxychloroquine exhibits the highest binding energy (-2.85 kcal/Mol). In terms of flavonoids Tetramethoxyflavone exhibit, the lowest binding energy (-4.91 kcal/Mol) and Gallocatechin exhibits the highest binding energy (-2.88). Moreover, Vitamin D showed the lowest binding energy (-5.52 kcal/Mol) and Vitamin C the lowest binding energy (-2.95 kcal/Mol).

| | **Antiparasitic** | **Binding energy (kcal/Mol)** | **2D interaction** | **Interface amino acids** | **Type of interactions** |
|---|---|---|---|---|---|
| 1 | Ivermectin B1a ($C_{48}H_{74}O_{14}$), 6321424 | -9.16 | 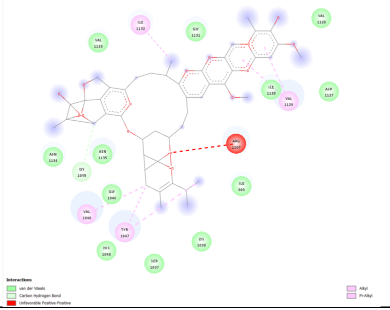 | 1Arg, 2Asn, 1Asp, 2Gly, 1His, 3Ile, 2Lys, 1Ser, 1Tyr, 4Val | 4Alk, 1CHB, 2Pi-Alk, 1UPP, 12VW |
| 2 | Ivermectin B1b ($C_{47}H_{72}O_{14}$), 6321425 | -8.86 | 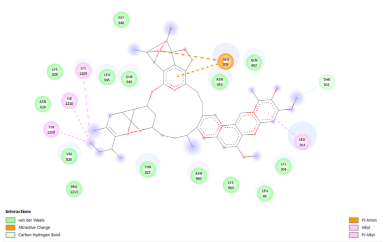 | 3Asn, 2Gln, 1Glu, 1Gly, 1Ile, 3Leu, 4Lys, 1Pro, 2Thr, 1Tyr, 1Val | 2AC, 3Alk, 1CHB, 1Pi-Alk, 14VW |
| 3 | Hydroxychloroquine ($C_{18}H_{26}ClN_3O$), 3652 | -2.85 | 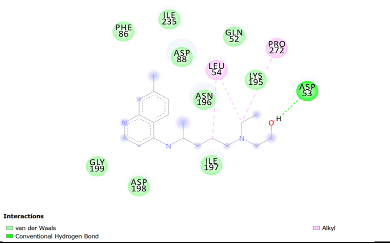 | Asn, 3Asp, Gln, Gly, 2Ile, Leu, Lys, Phe, Pro | 2Alk, 1HB, 9VW |

| | **Flavonoids** | **Binding energy (kcal/Mol)** | **2D interaction** | **Interface amino acids** | **Type of interactions** |
|---|---|---|---|---|---|
| 1 | Tetramethoxyflavone ($C_{19}H_{18}O_6$), 471721 | -4.91 | 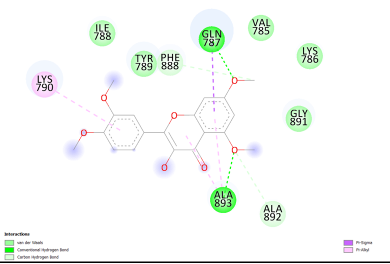 | 2Ala, 1Gln, 1Gly, 1Ile, 2Lys, 1Phe, 1Tyr, 1Val | 2CHB, 2HB, 3Pi-Alk, 1Pi-Sig, 5VW |

| | | | | | |
|---|---|---|---|---|---|
| 2 | Herbacetin ($C15H10O7$), 5280544 | -4.74 | 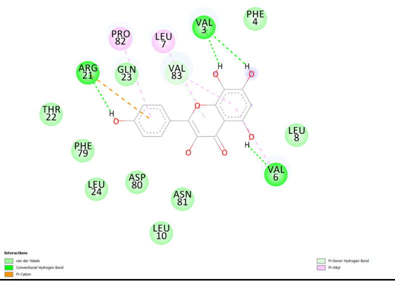 | 1Arg, 1Asn, 1Asp, 1Gln, 4Leu, 2Phe, 1Pro, 1Thr, 3Val | 4HB, 4Pi-Alk, 1Pi-Cat, 1Pi-DHB, 9VW |
| 3 | Gallocatechin ($C15H14O7$), 65084 | -2.88 | 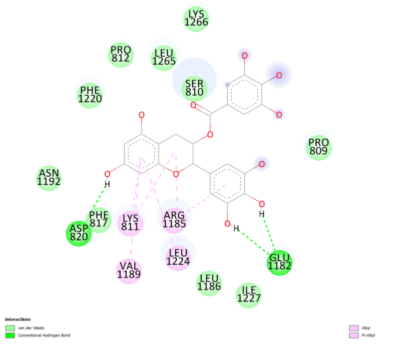 | 1Arg, 1Asn, 1Asp, 1Glu, 1Ile, 3Leu, 2Lys, 1Ser, 2Phe, 2Pro, 1Val | 2Alk, 3HB, 4Pi-Alk, 10VW |
| | | | | | |
| | **Vitamins** | **Binding energy (kcal/Mol)** | **2D interaction** | **Interface amino acids** | **Type of interactions** |
| 1 | Vitamin D ($C27H44O$), 5280795 | -5.52 | 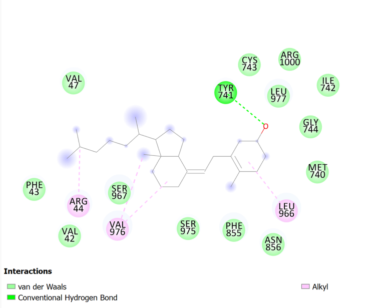 | 2Arg, Asp, Cys, Gly, Ile, 2Leu, Met, 2Phe, 2Ser, Tyr, 3Val | 3Alk, 1HB, 12VW |
| 2 | Vitamin C ($C6H8O6$), 54670067 | -2.95 | 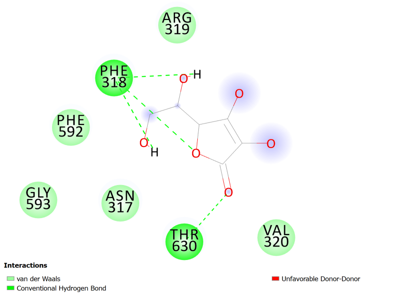 | Arg, Asn, Gly, 2Phe, Thr, Val | 4HB, 5VW |

**Table S4**: A comparative analysis between SARS-CoV-2 RdRP binding affinities and spike protein binding affinities in antivirals which primarily target viral RdRP. These comparisons include binding energy, the specific amino acids that are interacting with the drug, and the types of bonds formed during this interaction.

| | **Name and PubChem ID** | **Bound molecule** | **Binding Energy (kcal)** | **2D Interaction Plot** | **Interface Amino Acid** | **Type of Interactions** |
|---|---|---|---|---|---|---|
| 1 | Beclabuvir ($C_{36}H_{45}N_5O_5S$), 49773361 | RdRp | -5.63 | 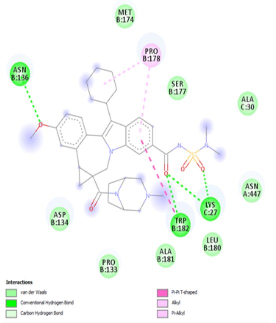 | 2Ala, 2Asn, Asp, Leu, Lys, Met, 2Pro, Ser, Trp | 4HB, 2Pi-Alk, 1Pi-Pi-T, 8VW |
| | | Spike | -6.64 | 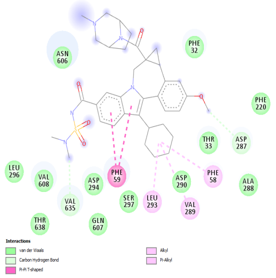 | Ala, Asn, 3Asp, Gln, 2Leu, 4Phe, Ser, 2Thr, 3Val | 2Alk, 2CHB, 1Pi-Alk, 2Pi-Pi-T; 12VW |
| 2 | Galidesivir ($C_{11}H_{15}N_5O_3$), 10445549 | RdRp | -4.38 | 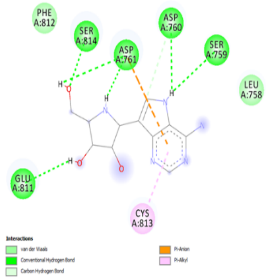 | 2Asp, Cys, Glu, Leu, Phe, 2Ser | 1CHB, 6HB, 1Pi-Alk, 1Pi-Ani, 2VW |
| | | Spike | -3.11 | 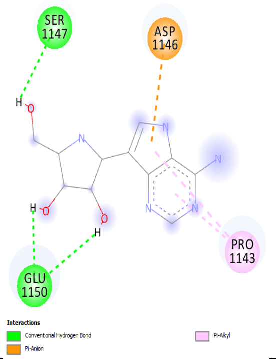  | Asp, Glu, Pro, Ser | 3HB, 2Pi-Alk, 1Pi-An |

| | | | | | | |
|---|---|---|---|---|---|---|
| 3 | Ribavirin (C8H12N4O5), 37542 | RdRp | -4.2 | 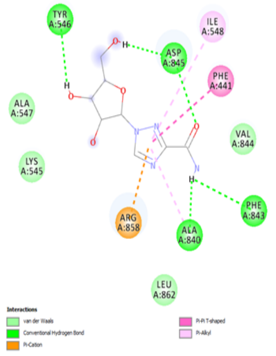 | 2Ala, Arg, Asp, Ile, Leu, Lys, 2Phe, Val | 5HB, 2Pi-Alk, 1Pi-Cat, 1Pi-Pi, 4VW |
| | | Spike | -3.29 | 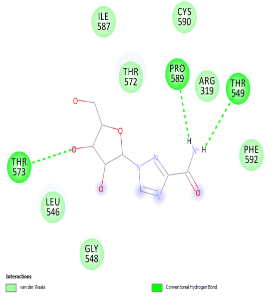 | Arg, Cys, Gly, Ile, Leu, Phe, Pro, 3Thr | 3HB, 7VW |
| 4 | Favipiravir (C5H4FN3O2), 492405 | RdRp | -3.09 | 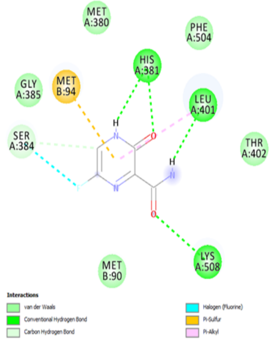 | Gly, His, Leu, Lys, 3Met, Phe, Ser, Thr | 1CHB, 1Hal, 4HB, 1Pi-Alk, 1Pi-Sul, 5VW |
| | | Spike | -2.94 | 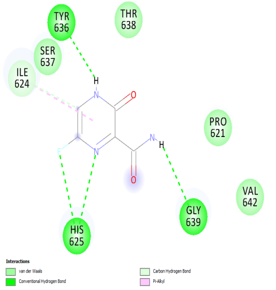 | Gly, His, Ile, Pro, Ser, Thr, Tyr, Val | 1CHB, 4HB, 1Pi-Alk, 4VW |

| | | | | | | |
|---|---|---|---|---|---|---|
| 5 | Sofosbuvir ($C22H29FN3O9P$), 45375808 | RdRp | -2.95 | 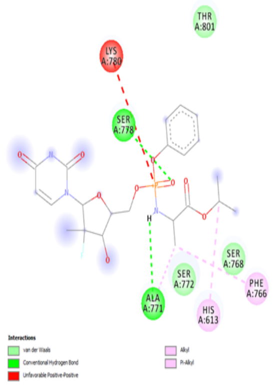 | Ala, His, Lys, Phe, 3Ser, Thr | 1Alk, 2HB, 2Pi-Alk, 3VW, 1UPP |
| | | Spike | -3.7 | 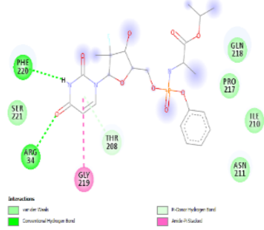 | Arg, Asn, Gln, Gly, Ile, Phe, Pro, Ser, Thr | 1APS, 2HB, 1Pi-DHB, 5VW |
| 6 | Tenofovir ($C9H14N5O4P$), 464205 | RdRp | -2.34 | 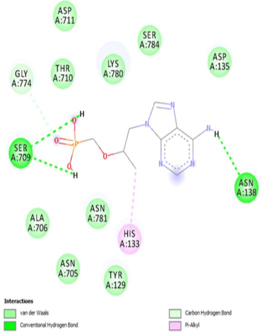 | Ala, 3Asn, 2Asp, Gly, His, Lys, 2Ser | 1CHB, 3HB, 1Pi-Alk, 9VW |
| | | Spike | -2.75 | 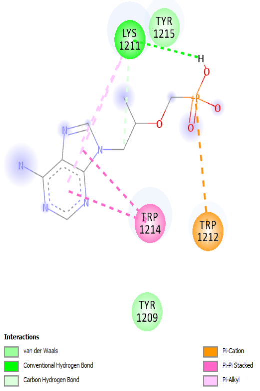 | Lys, 2Trp, 2Tyr | 1CHB, 1HB, 2Pi-Alk, 1Pi-Cat, 2Pi-Pi-Stk, 2VW |

| | | | | | | |
|---|---|---|---|---|---|---|
| 7 | Remdesivir ($C_{27}H_{35}N_6O_8P$), 121304016 | RdRp | -1.28 | 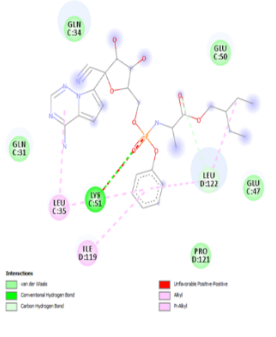 | 2Gln, 2Glu, Ile, 2Leu, Lys, Pro | 1Alk, 1CHB, 1HB, 4Pi-Alk, 5VW, 1UPP |
| | | Spike | -2.65 | 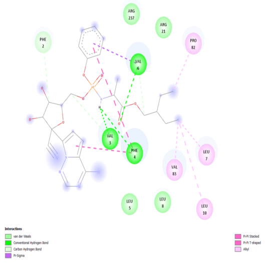 | 2Arg, 4Leu, 2Phe, 1Pro, 3Val | 4Alk, 1CHB, 3HB, 1Pi-Pi-T, 1Pi-Pi-Stk, 1Pi-Sig, 4VW |

# Figure S1

A

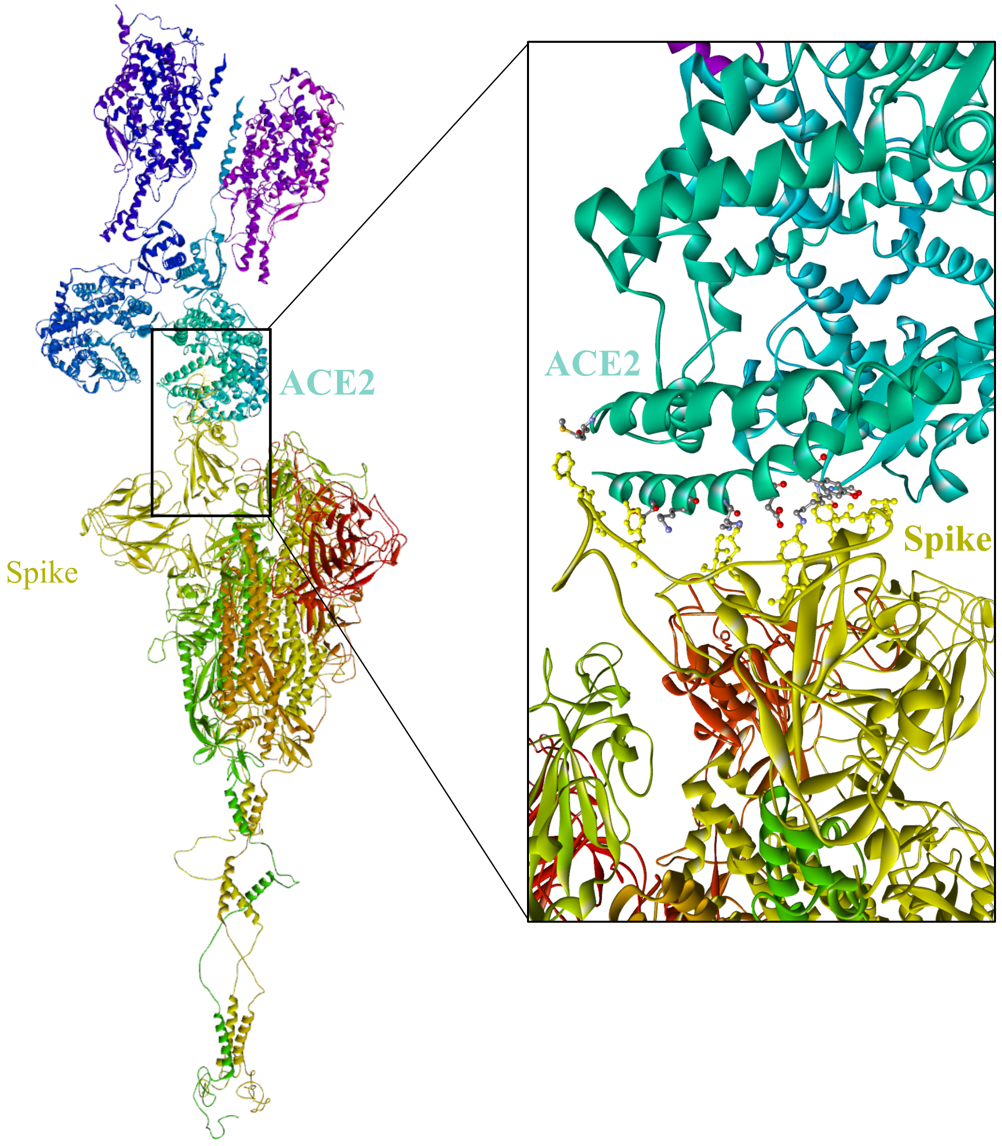
ACE2
Spike
ACE2
Spike

B

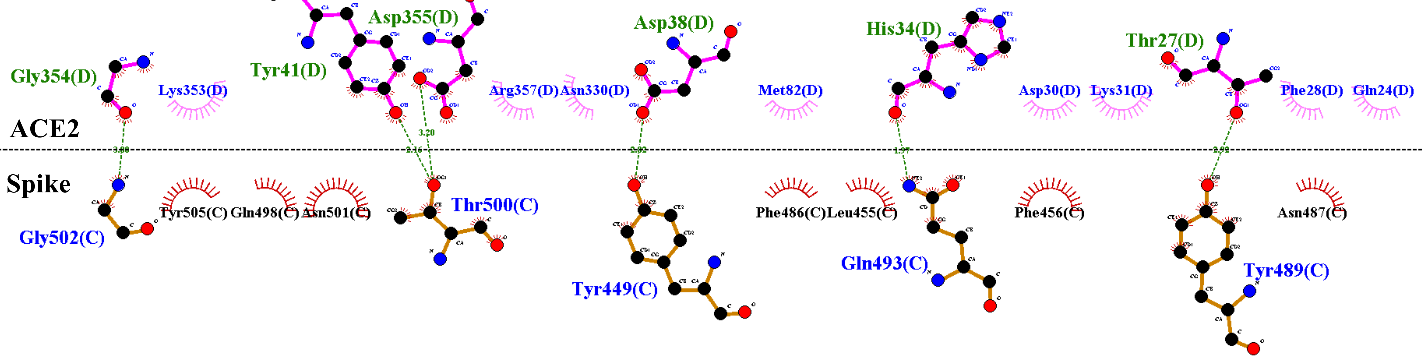
Gly354(D)
Lys353(D)
Tyr41(D)
Asp355(D)
Arg357(D)
Asn330(D)
Asp38(D)
Met82(D)
His34(D)
Asp30(D)
Lys31(D)
Thr27(D)
Phe28(D)
Gln24(D)
ACE2
Spike
Gly502(C)
Tyr505(C)
Gln498(C)
Asn501(C)
Thr500(C)
Tyr449(C)
Phe486(C)
Leu455(C)
Gln493(C)
Phe456(C)
Asn487(C)
Tyr489(C)

# Figure S2

**A**

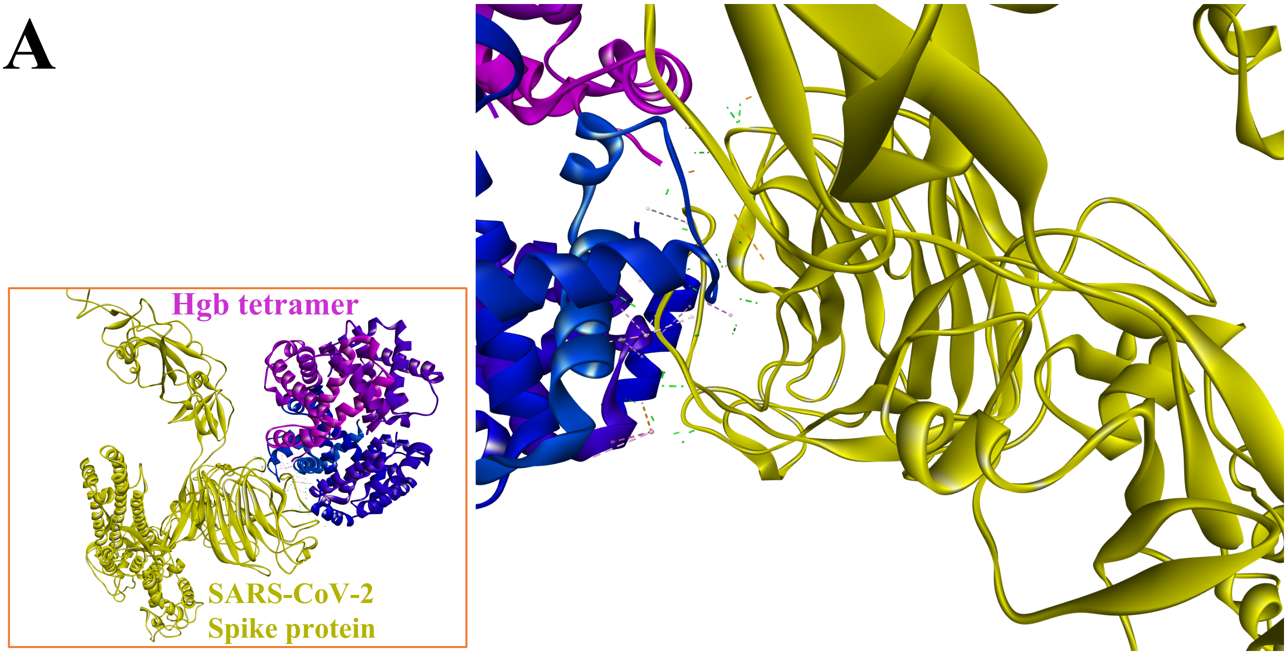
Hgb tetramer
SARS-CoV-2
Spike protein

**B**

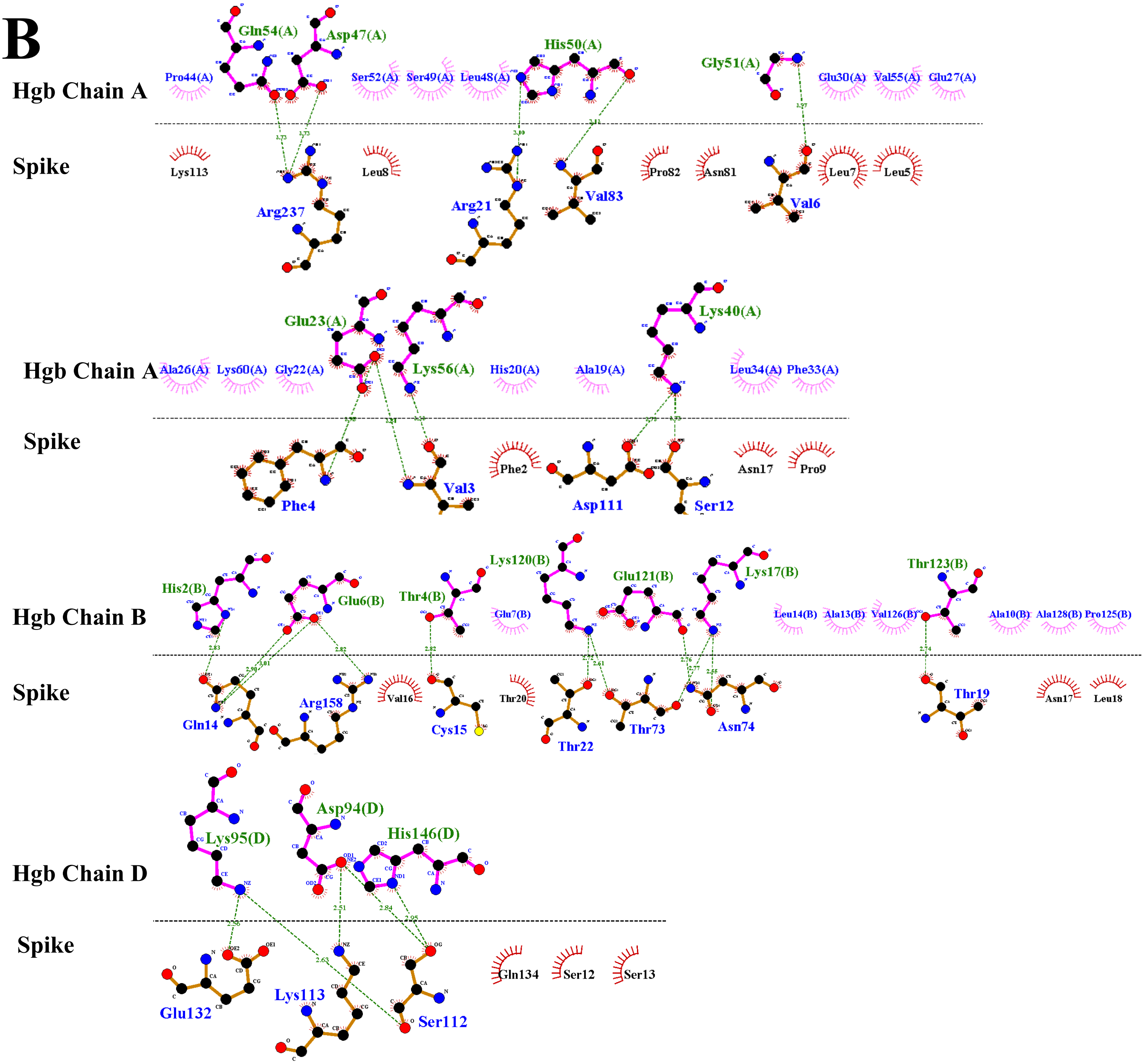
Hgb Chain A
Spike
Gln54(A)
Asp47(A)
His50(A)
Gly51(A)
Pro44(A)
Ser52(A)
Ser49(A)
Leu48(A)
Glu30(A)
Val55(A)
Glu27(A)
Lys113
Leu8
Arg237
Arg21
Val83
Pro82
Asn81
Leu7
Leu5
Val6
Hgb Chain A
Spike
Glu23(A)
Lys40(A)
Ala26(A)
Lys60(A)
Gly22(A)
Lys56(A)
His20(A)
Ala19(A)
Leu34(A)
Phe33(A)
Phe4
Val3
Phe2
Asp111
Ser12
Asn17
Pro9
Hgb Chain B
Spike
His2(B)
Glu6(B)
Thr4(B)
Lys120(B)
Glu121(B)
Lys17(B)
Thr123(B)
Glu7(B)
Leu14(B)
Ala13(B)
Val126(B)
Ala10(B)
Ala128(B)
Pro125(B)
Gln14
Arg158
Val16
Cys15
Thr20
Thr22
Thr73
Asn74
Thr19
Asn17
Leu18
Hgb Chain D
Spike
Lys95(D)
Asp94(D)
His146(D)
Glu132
Lys113
Ser112
Gln134
Ser12
Ser13

# Figure S3

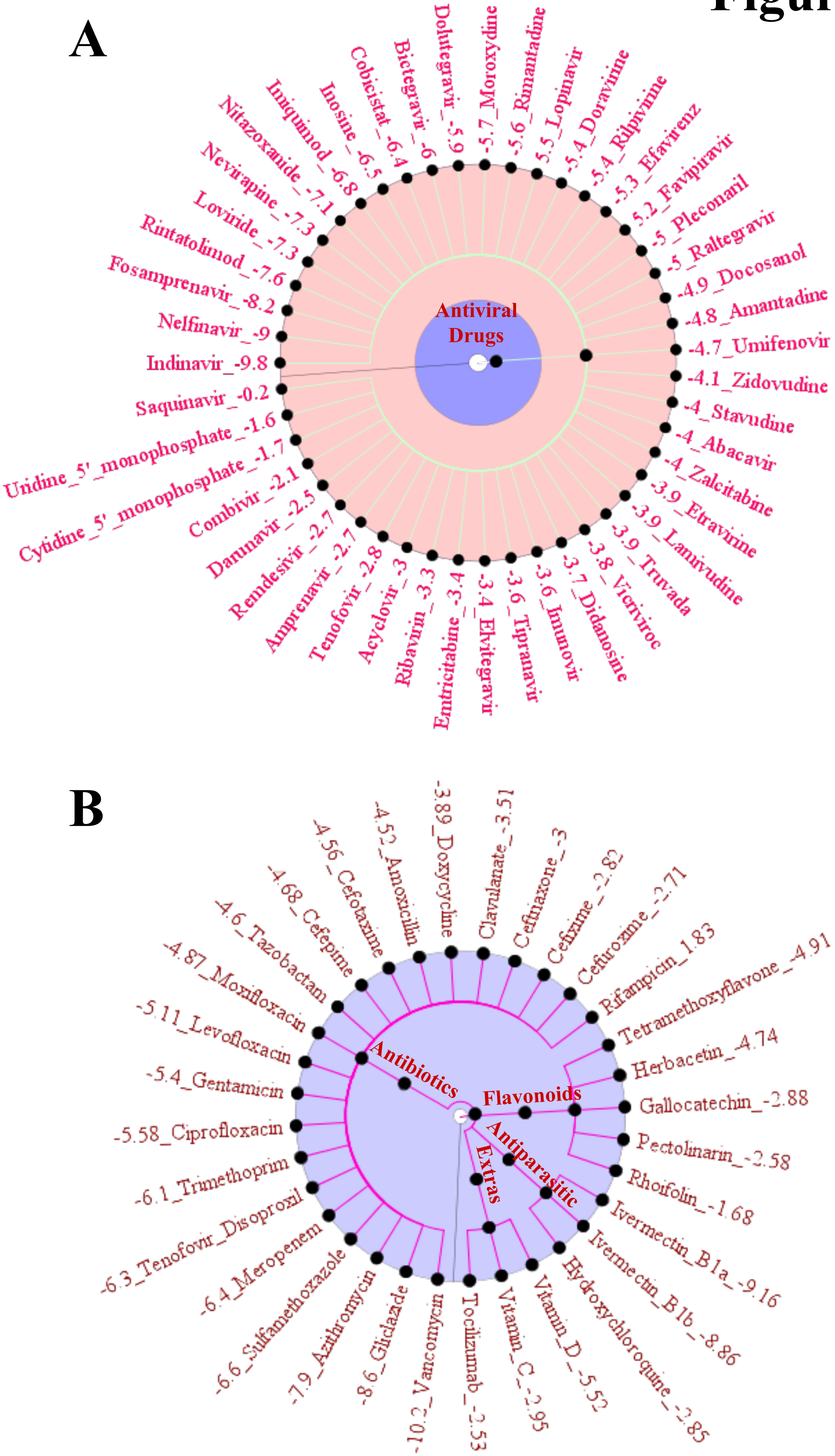
A
Antiviral Drugs
Indinavir_-9.8
Nelfinavir_-9
Fosamprenavir_-8.2
Rintatolimod_-7.6
Loviride_-7.3
Nevirapine_-7.3
Nitazoxanide_-7.1
Imiquimod_-6.8
Inosine_-6.5
Cobicistat_-6.4
Bictegravir_-6
Dolutegravir_-5.9
-5.7_Moroxydine
-5.6_Rimantadine
-5.5_Lopinavir
-5.4_Doravirine
-5.4_Rilpivirine
-5.3_Efavirenz
-5.2_Favipiravir
-5_Pleconaril
-5_Raltegravir
-4.9_Docosanol
-4.8_Amantadine
-4.7_Umifenovir
-4.1_Zidovudine
-4_Stavudine
-4_Abacavir
-4_Zalcitabine
-3.9_Etravirine
-3.9_Lamivudine
-3.9_Truvada
-3.8_Vicriviroc
-3.7_Didanosine
-3.6_Imunovir
-3.6_Tipranavir
-3.4_Elvitegravir
Emtricitabine_-3.4
Ribavirin_-3.3
Acyclovir_-3
Tenofovir_-2.8
Amprenavir_-2.7
Remdesivir_-2.7
Darunavir_-2.5
Combivir_-2.1
Cytidine_5'_monophosphate_-1.7
Uridine_5'_monophosphate_-1.6
Saquinavir_-0.2
B
Antibiotics
Flavonoids
Antiparasitic
Extras
-3.89_Doxycycline
Clavulanate_-3.51
Ceftriaxone_-3
Cefixime_-2.82
Cefuroxime_-2.71
Rifampicin_1.83
Tetramethoxyflavone_-4.91
Herbacetin_-4.74
Gallocatechin_-2.88
Pectolinarin_-2.58
Rhoifolin_-1.68
Ivermectin_B1a_-9.16
Ivermectin_B1b_-8.86
Hydroxychloroquine_-2.85
Vitamin_D_-5.52
Vitamin_C_-2.95
Tocilizumab_-2.53
-10.2_Vancomycin
-8.6_Gliclazide
-7.9_Azithromycin
-6.6_Sulfamethoxazole
-6.4_Meropenem
-6.3_Tenofovir_Disoproxil
-6.1_Trimethoprim
-5.58_Ciprofloxacin
-5.4_Gentamicin
-5.11_Levofloxacin
-4.87_Moxifloxacin
-4.6_Tazobactam
-4.68_Cefepime
-4.56_Cefotaxime
-4.52_Amoxicillin
